# Aspirin versus low-molecular-weight heparin for venous thromboembolism prophylaxis: a trial sequential analysis

**DOI:** 10.1097/JS9.0000000000001003

**Published:** 2023-12-14

**Authors:** Kuo-Chuan Hung, I-Wen Chen, Ping-Hsin Liu

**Affiliations:** a Department of Anesthesiology, Chi Mei Medical Center, Tainan City; b Department of Anesthesiology, Chi Mei Medical Center, Liouying, Tainan City; c Department of Anesthesiology, E-Da Dachang Hospital, I-Shou University, Kaohsiung City, Taiwan

*Dear Editor,*

We read with great interest the recent article by Meng *et al*.^1^ titled ‘The role of aspirin versus low-molecular-weight heparin for venous thromboembolism prophylaxis after total knee arthroplasty: a meta-analysis of randomized controlled trials’ published in the *International Journal of Surgery*. The authors performed a meta-analysis of six randomized controlled trials (RCTs) comparing aspirin to low-molecular-weight heparin (LMWH) for venous thromboembolism (VTE) prophylaxis after total knee arthroplasty (TKA)^1^. They found that aspirin was associated with a significantly higher risk of VTE compared to LMWH^1^. We would like to raise some concerns regarding this meta-analysis.

Firstly, the rationale behind using aspirin for VTE prophylaxis after TKA is that it is simple, affordable, and does not require monitoring compared to LMWH injections. Hence, aspirin may improve post-discharge adherence. The authors should comment on this important benefit of aspirin in the discussion. Secondly, although the primary outcome of the VTE rate showed a statistically significant difference favoring LMWH, this evidence may still be inadequate. Standard meta-analyses do not account for sparse data and repeated testing of accumulated data, which can increase the risk of false positive findings. Trial sequential analysis (TSA) can overcome these limitations by calculating the required information size and providing adjusted confidence intervals^2,3^. As TSA was not performed in this originally published meta-analysis^1^, the significant result could represent a false positive finding.

To further assess the evidence, we conducted TSA on the extracted VTE data from the originally published meta-analysis^1^. The TSA was conducted using TSA viewer version 0.9.5.10 Beta (www.ctu.dk/tsa), with an alpha of 5%, power of 80%, and relative risk reduction of 20%. The TSA-adjusted 95% confidence intervals were 0.96–2.22, and the *z*-curve did not cross the trial sequential monitoring boundary (Fig. 1), suggesting that the evidence is still inconclusive. Accordingly, more trials are needed to reach the required information size of 18 457 patients before we can safely conclude aspirin is inferior for VTE prevention compared to LMWH. Therefore, while the originally published meta-analysis may favor LMWH^1^, it remains premature to recommend against aspirin for TKA thromboprophylaxis considering its other benefits. More adequately powered RCTs comparing these two agents are still required.

**Figure 1:**
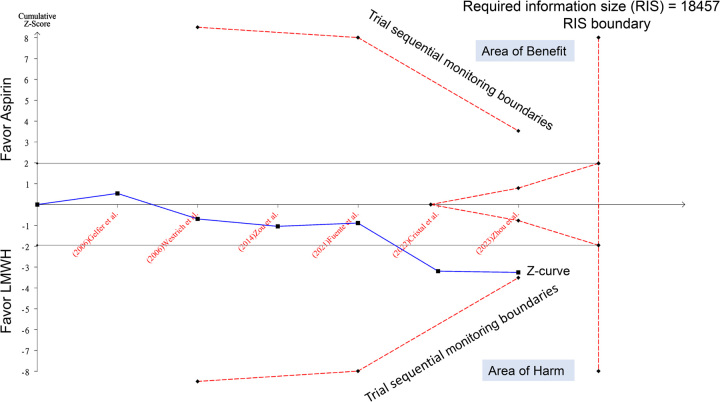
Trial sequential analysis of venous thromboembolism (VTE) rate in total knee arthroplasty patients receiving aspirin versus low-molecular-weight heparin (LMWH). The required information size of 18 457 patients was calculated using an alpha of 5%, power of 80%, and a relative risk reduction of 20%. The cumulative *z*-curve (blue line) does not cross the trial sequential monitoring boundary (red inward sloping lines) and does not reach the required information size (RIS) (vertical red line), indicating that the evidence for a risk difference between aspirin and LMWH is still inconclusive. More studies are required before a definitive conclusion can be reached.

## Ethical approval

Not applicable.

## Consent

Not applicable.

## Sources of funding

Not applicable.

## Author contribution

K.-C.H. and P.-H.L.: wrote the main manuscript text; I.-W.C.: prepared Figure 1. All authors read and approved the final version of the manuscript.

## Conflicts of interest disclosure

The authors declare no conflicts of interest.

## Research registration unique identifying number (UIN)

Not applicable.

## Guarantor

Kuo-Chuan Hung.

## Data availability statement

The datasets used and/or analyzed in the current study are available from the corresponding author upon reasonable request.

## Provenance and peer review

This paper was not invited.
